# Viscoelastic Properties of Cell Structures Manufactured Using a Photo-Curable Additive Technology—PJM

**DOI:** 10.3390/polym13111895

**Published:** 2021-06-07

**Authors:** Tomasz Kozior, Czesław Kundera

**Affiliations:** Department of Manufacturing Technology and Metrology, Kielce University of Technology, Al. Tysiąclecia Państwa Polskiego 7, 25-314 Kielce, Poland; kundera@tu.kielce.pl

**Keywords:** PJM, 3D printing, rheology, cellular structures, quality

## Abstract

This research paper reviews the test results involving viscoelastic properties of cellular structure models made with the PolyJet Matrix—PJM additive technology. The designed test specimens were of complex cellular structure and made of three various photo-curable polymer resin types. Materials were selected taking into account the so-called “soft” and “tough” material groups. Compressive stress relaxation tests were conducted in accordance with the recommendations of standard ISO 3384, and the impact of the geometric structure shape and material selection on viscoelastic properties, as well as the most favorable geometric variants of the tested cellular structure models were determined. Mathematica and Origin software was used to conduct a statistical analysis of the test results and determine five-parameter functions approximating relaxation curves. The most favorable rheological was adopted and its mean parameters determined, which enables to match both printed model materials and their geometry in the future, to make a component with a specific rheological response. Furthermore, the test results indicated that there was a possibility of modelling cellular structures within the PJM technology, using support material as well.

## 1. Introduction

3D printing technologies have been known for over 40 years, which is why their current stage is very advanced. New materials and increasing both the process capabilities of the machines and accuracy of produced models are currently the subject of intensive work. New measurement technologies that enable assessment of hard-to-analyses models of complex shapes [1], produced using 3D printing techniques [2,3] are being dynamically developed. Furthermore, owing to the application of appropriate production process control for controlling process parameters, there is a possibility to make models with mechanical properties that can be adjusted from machine software level, through setting printing parameters [4,5,6,7,8,9,10], similarly to cases of using conventional technologies [11]. Due to the fact that 3D printing technologies differ both in terms of input material condition and layer binding method, the number of process parameters is variable, but the main ones include printing direction, layer thickness, laser speed and power, hatch distance, build chamber temperature etc. Mechanical properties and the dimension and shape accuracy of additive-shaped models is currently at such a high level that 3D printing technologies are used in such industries as foundry [12,13], jewelry, aviation and automotive [14], food and beverage, architecture, medicine [15,16,17] etc.

The development of additive technologies entailed new process possibilities, such as the option to build new composites [18,19,20] and cellular structures applied in numerous fields of industry, also in medicine. Cellular structures, owing to their very good vibration damping properties, can be used in many industrial and medical solutions, such as the medical “da Vinci” robot, which utilizes 3D printing to build instrumentation, such as shock absorbers, dampers, hooks, etc.

Robotics, as a highly variable industrial sector with a great degree of prototype model application is one of the main consumers of 3D printing technologies. It is particularly visible in the case of building all kinds of actuators, soft robotics, as well as housing elements and covers. The use of 3D and 4D printing technologies in robotics primarily involves applying methods that print using plastic-based materials and smart materials [21], such as composites with carbon and glass fibers, as well as rubber-based materials. Many examples of the use of 3D/4D printing technology in robotics have been discussed in numerous research work [22,23,24,25,26]. 

Cellular structures can currently be made using 3D printing technologies, although bear in mind that not every additive technology is suitable for that purpose. However, in the case of photo-curing technologies, such as PolyJet Matrix, it is possible to print a fully finished structure. PJM enables printing based on several materials at the same time, which makes it possible to build a tough matrix and soft filling [27]. This is impossible in the case of such technologies as [28] selective laser melting (SLM) [6,29], fused deposition modeling (FDM), stereolithography (SLA), laminated object manufacturing (LOM) etc. The application of several materials within the PJM technology results in obtaining a single model with various physico-chemical properties at different model areas, without the need to fill the model at a later stage. Currently, there are already available publications, the authors of which used 3D printing technologies for building cellular structures [30,31,32,33,34,35,36,37], with some of them discussed below. 

In [30], the authors conducted experimental tests of cellular structures made of the FullCure M840 photopolymer and silicone rubber as pore filler. The obtained multi-cellular structures in the form of cuboidal specimens were studied using standard compression tests on a universal testing machine, in static and dynamic conditions. The obtained compressive test results were used to assess the behavior of cellular structures under low (quasi-static) and high relative strains. The ability of the studied structure to absorb energy was also evaluated based on this data.

Vesenjak et al. [31] studied the impact of the shape of a cell within a given structure on its behavior in the course of uniaxial compression tests. Cellular structures with round and square cells were made using the PA 12 polyamide and laser sintering. The experimental test results indicated better properties in structures with round-shape cells, compared to structures with square cells.

Experiments involving cellular structures made of plastics were also described by Wyatt et al. [32]. The authors compared the results of experimental static compression test results for cellular structure model specimens with the results of numerical calculations. In [33] Vesenjak et al. described the impact of a pore (cell) filler material on the energy absorption ability of a cellular structure. They tested the behavior of cellular structures made of aluminum alloy and polymer material, without pore filling and with pores filled with silicone rubber under quasi-static and dynamic compressive load conditions. The results showed that the pore filler improved energy absorption capacity. Furthermore, the findings included the improvement and stabilization of the dynamic response in brittle cellular structures. 

An overview of materials used to build auxetic cellular structures is shown in paper [34]. The authors presented a wide range of materials, their mechanical properties, applications, most common profiles (with internal shapes) and manufacturing methods. Furthermore, the article describes the further development potential for cellular structures. A study of the mechanical properties of regular 2D cellular structures were reviewed in publication [35]. The original topologies of regular 2D cellular structures developed by the authors were built using the Fused Deposition Modeling (FDM) additive modelling method, and then subjected to compression tests. Test specimens were prepared from three commercially available polymer materials—ABSplus, Nylonu 12 and PC-10. Based on the obtained results, it was concluded that specimens with higher rigidity exhibit more effective energy absorption properties.

When analyzing the presented test results published in the aforementioned scientific articles, it seems that the key in terms of building cellular structure models is to define their behavior under continuous load over time. This primarily applies to two types of tests, namely, relaxation and creep, therefore, tests that reflect rheological properties. Moreover, it should be emphasized that these properties are omitted by machine and material manufacturers in official documents, which specify mechanical properties. 

Compressive stress relaxation tests within a uniaxial compression tests on models made using various (selected) polymer materials in FDM, PJM and SLS technologies were shown in selected publications [4,38,39,40,41,42]. These tests showed that mechanical properties of the prepared models depended on preset process parameters, such as printed layer thickness and model orientation relative to the building platform, so-called “printing direction”, as well as SLS laser operating parameters.

Bochnia in [41] presented the results of stress relaxation tests for digital materials used in 3D printing technology—PJM. Cylindrical samples were used for the tests, which were made with two preset printing angles on the building platform. The complex 5-parameter Maxwell-Wiechert model was also used to assess the rheological properties. Both material: DM_8515_Grey35 and DM_9895_Shore95 were tested, which belong to the materials obtained by mixing two base materials: soft Tango and tough Vero. The test results showed that these materials are characterized by a high anisotropy of mechanical properties depending on the printing direction and that the 5-parameter rheological model with a very good approximation allows the stress relaxation curve to be approximated.

The preliminary results of the authors’ research carried out for the VeroWhite and FullCure 720 base materials are described in work [42]. The tests carried out for cylindrical samples with a diameter of 13 mm and a height of 6.3 mm also took into account the influence of the print direction (0°, 45° and 90°) on the rheological properties. The results of the research showed that the PJM technology is characterized by a high anisotropy of mechanical properties depending on the direction of layering of the model material in the case of FullCure 720 material. In the case of VeroWhite material, no significant changes in stress relaxation coefficients in relation to the print direction were noticed. Moreover, the stress relaxation coefficients were determined, where a 5-parameter rheological model was also used.

Tests of cellular structure models printed using acrylic resins (FullCure 720 and VeroWhite photopolymers) in the PJM technology are reviewed in the original work [43]. The results indicated that combining these two materials into cellular structure with a preset geometry, with a simultaneously printed filling, enabled obtaining models of varying mechanical properties and that it was possible to control the process, in order to obtain models of required rheological response and appropriate damping properties.

In [44] Gibson and Ashby evidenced that the density of a honeycomb structure was identified as one of the dominating factors impacting its mechanical properties. 

Based on the literature review and the original test results, it was possible to conclude that cellular structures made with additive technologies exhibit interesting mechanical properties that determine their actual application in various industries. 

The authors of this paper presented the results of stress relaxation tests for different cellular structure models, made from three types of photo-curable resins using the PJM technology. Experimental relaxation curves were used as a base to determine the relative compressive stress reduction and identify the parameters of the rheological model adopted to describe the viscoelastic properties of studied cellular structure models.

The models adopted for the tests were designed so that it was possible to relate the results obtained for a single structure to more complex models consisting of many identical cells, distributed along three axes X, Y and Z. Such a research approach may lead to the test results being used in such fields as mechanical engineering, automation and robotics, medicine and many others.

## 2. Materials and Methods

### 2.1. Materials

Three types of photo-curable resins (Stratasys, Eden Prairie, MN, USA) applied in the PJM technology: FullCure 720 (FC 720), VeroWhite (VW) and FullCure 705 (FC 705) were used to construct the models. The first two are model materials that belong to a group of so-called tough materials, with a Shore A hardness of over 80. The third material is support type, which exhibits elastic properties, therefore, it was used to fill the structures created in the course of the tests. Table 1, Table 2 and Table 3 show the chemical composition of materials used to construct specimen models. 

### 2.2. PJM Technology

**Figure 1:**
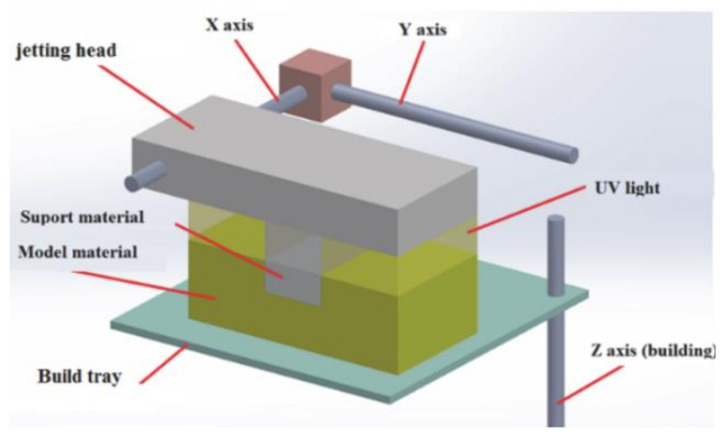
The process of producing models in a 3D printing technology—PJM.

Test specimens were made using a Connex 350 machine by Stratasys, which operates using the PolyJet Matrix (PJM) liquid polymer resin photo-curing technology. The Connex 350 printer is one of the most accurate 3D printers that enables building models in the High-Quality mode, with a layer thickness of 0.016 mm. The PJM technology involves spraying liquid polymer resins to areas of currently built layer, which corresponds to the model cross-section, followed by the initialization of the polymerization process using a lamp, as a UV light source. As a result, the applied layer is cured and combined with the previously formed one. Figure 1 [43] shows a PJM technology process scheme.

All specimen models were designed using SolidWorks software (Dassault Systèmes SolidWorks Corp., Waltham, MA, USA), and then saved as STL files in the so-called adapted save mode, with the parameters of deviation tolerance of 0.01, and angular tolerance of 5°. Next, the STL files were subjected to approximation verification using Magics 15.0 (Materialise, Belgium). After verification, digital models of the specimens were arranged on a virtual 3D printer platform. Models were made using a High-Quality mode and layer thickness of 0.016 mm.

### 2.3. Specimen Preparation

**Figure 2:**
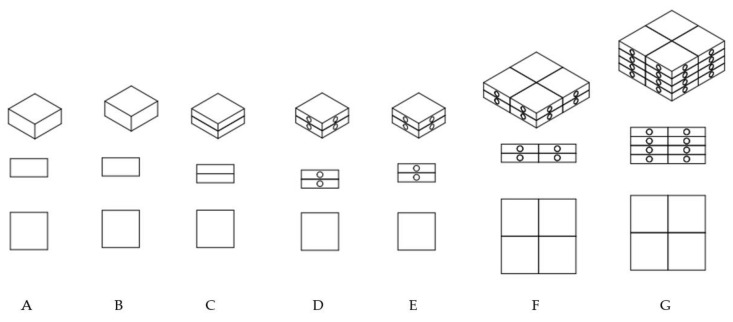
Test specimen model shapes, A–G samples type.

**Figure 3:**
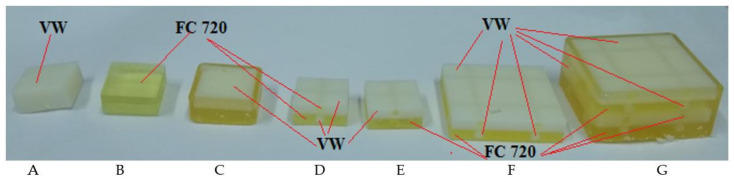
View of printed specimen models using a Connex printer, A–G samples type.

Figure 2 shows designed specimen models, while Figure 3 their finished physical models after printing and before cleaning process. The process of removing the support material applies only to external model surfaces. Whereas the filling of their interior was used as so-called “soft” support material in building a given specimen model structure. The dimensions of the samples A–E are 13 mm × 13 mm and 6.3 mm (high) result from the fact that our previously conducted test results were carried out for samples with a diameter of 13 mm and a height of 6.3 mm in accordance with the ISO 3384 standard. Results from this publication, can be used in the construction of other modular models with complex geometry.

All test specimens were in the shape of a cuboid with the following outer dimensions:

- Specimens A–E: base with 13 × 13 mm sides and a height of 6.3 mm.
- Specimens F and G: base with 26 × 26 mm sides and a height of 6.3 mm and 12.6 mm, respectively.

Specimens designated as A and B (Figure 2 and Figure 3) were made using model materials, respectively, VeroWhite (VW) and FullCure 720 (FC 720) resin.

The specimen designated as C was made using model materials (VW and FC 720) in the form of two combined cuboids, both with a height of 3.15 mm. Specimen D, similarly to specimen C was made using FC 720 and VW, and additionally, its each half had two cylindrical channels with a diameter of 2 mm, perpendicular to the axis, which were alternately filled with opposite model materials. The shape and dimensions of specimen E were identical to that of specimen D, except that the internal cylindrical channels were filled with support material FullCure 705 (FC 705). Specimens designated as C, D and E represent basic cells of complex structure that can be used for constructing multi-cellular structures. The next specimens, designated as F and G, can be examples of such multi-cellular structures with a relatively simple structure. Specimen F is a combination of four basic cells designated as specimen D. The last specimen, G, is a combination of two specimens F, which gives a total of 8 combined basic cells designated as specimen D.

### 2.4. Rheological Analysis

24 h after the specimens were cleaned using a water jet washer, the authors conducted rheological tests in the form of relaxation tests involving stresses determined during the uniaxial compression test. Specimens were fixed between universal testing machine discs, and then compressed at a rate of 1 mm/s, to a strain value of 10% of the specimen height (as per standard ISO 3384-1:2019 Rubber, vulcanized or thermoplastic—Determination of stress relaxation in compression—Part 1: Testing at constant temperature). Next, for a period of 60 min, the specimens were compressed while maintaining a constant strain value (10%—0.63 mm). After taking the measurements, the authors determined the stress relaxation value as per Equation 1 and calculated standard deviation—SD (three samples for each type).

(1) $$\Delta \sigma =\frac{\sigma_{o}-\sigma_{t}}{\sigma_{o}}$$

where:

*σ_o_*—initial stress; *σ_t_*—stress after predetermined relaxation time t.

In the course of further description of relaxation measurements, the relative decrease in the compressive stress (1) will be called the stress relaxation index.

## 3. Results

**Figure 4:**
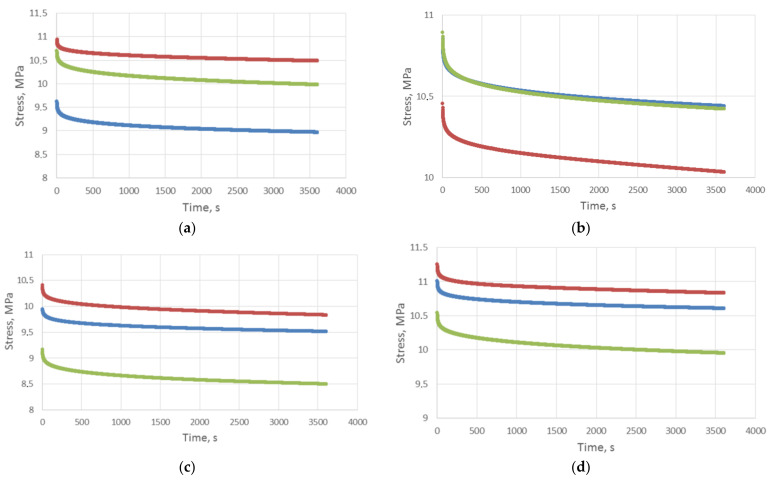

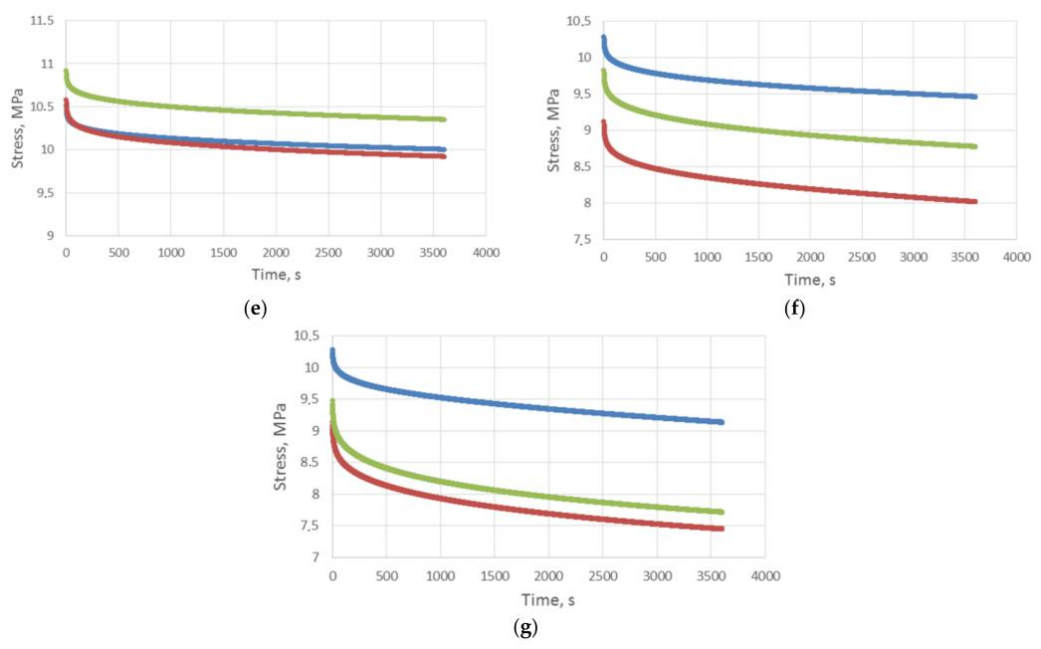
Relaxation characteristics of test specimens, (**a**) samples—A, (**b**) samples—B, (**c**) samples—C, (**d**) sampes—D, (**e**) samples—E, (**f**) samples—F, (**g**) samples—G.

Rheological tests covering specimen models were conducted using an Inspekt mini 3 kN universal testing machine (Hegewald and Peschke, Nossen, Germany). Microscopic surface analysis was conducted using a stereoscopic microscope by OLYMPUS. Figure 4 shows experimental characteristics of compressive stress relaxation for tested specimens (A–G), as in Figure 3.

The results of compressive stress relaxation indices calculations for each test specimen of a given structure (Figure 3) are shown in Table 4.

Table 4 shows mean value of the stress relaxation index determined on the basis of relationship (1) for all specimen types (A–G) and always for three (3) samples in each type (21 samples in total).

**Figure 5:**
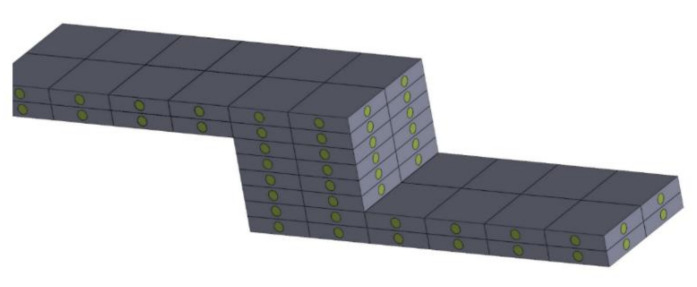
Model of a lever with a modular structure of studied cell structures.

As can be concluded based on the data in Table 4, the stress relaxation in the case of specimens A and B, made from VeroWhite and FullCure 720, respectively, is the highest for VeroWhite and amounts to 0.095. An interesting conclusion can be drawn from analyzing specimen C, which combines two mentioned materials, where the authors obtained relaxation at a level of VeroWhite. Specimen designated as D, with the dimensions and combination the same as specimen C, however, with internal structures in the form of intersecting cylindrical channels (openings) filled alternately with model materials, exhibited the least stress relaxation. It can be concluded that, in this case, the application of internal cellular structures results in the material being much more resistant to continuous loads over time. Whereas specimen D, compared to specimen C, recorded a stress relaxation lower by 25%. In the case of analyzing models made the same way as specimens D, but with an internal structure made of “soft” support material, it can be seen that stress relaxation increased by more than 54%, relative to specimen D. Such a significant value results from combining both tough material as the matrix and soft material as the filling. Specimens F and G that are an expansion of the aforementioned structure of specimen D, exhibit stress relaxation at a similar level (0.033 and 0.039), despite the fact that both were compressed until reaching a strain of 0.063 mm. The largest dispersion of the measurement results expressed by the standard deviation parameter is observed in the case of sample A, where SD—0.049. The lowest value of standard deviation was observed for samples marked with symbols B and F where SD—0.005. It can be concluded that the construction of small cellular structures and the application of both soft and tough materials reduces stress relaxation, as shown in model D. Furthermore, the application of repeated structural assemblies makes 3D models also more resistant to continuous loads over time. It seems that such a characteristic, arising from the conducted tests, may lead to conclusions that cellular structure models and their expansion through modular linking of structures can be greatly beneficial if they are used, for example, in robotics and automation as elements of grippers, levers or drive conveyors subjected to continuous loads over time. An example of a model with a cellular and modular structure is shown in Figure 5.

Less or more complex rheological models are used to describe the viscoelastic properties of materials [38,41,45,46,47]. The parameters of these models are determined experimentally.

Based on the compressive stress relaxation measurement (test) results, the authors approximated them mathematically using a five-parameter relaxation function in the following form (2):

(2) $$\sigma \left(t\right)=\sigma_{o}+\sigma_{1}e^{-\frac{t}{t_{1}}}+\sigma_{2}e^{-\frac{t}{t_{2}}}$$

where:

*t*_1_, *t*_2_—relaxation times.

*σ_o_*, *σ*_1_, *σ*_2_—partial stresses.

**Figure 6:**
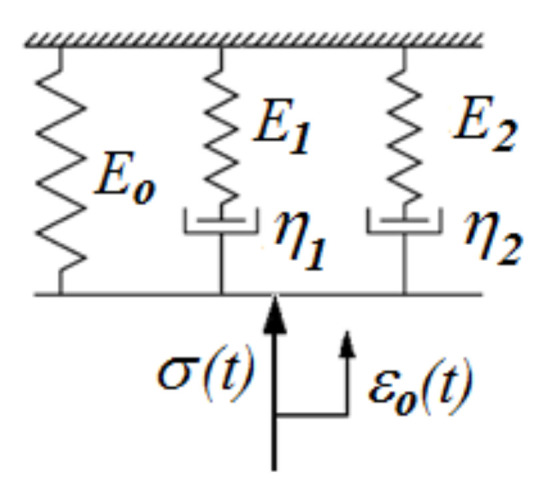
Five-parameter rheological model.

The process of matching the rheological process to the results obtained in the course of the research was conducted using the Origin software, utilizing an implemented Levenberg-Marquardt (L-M) method for approximating test results with a preset function, the five-parameter function in this case. Furthermore, this software was used to determine the R^2^ coefficient, which defines the degree of matching an approximated function to actual test results. The adopted stress relaxation function (2) is a response to applied step strain during the material relaxation test *σ*(*t*) = *ε_o_H*(*t*), described by the Maxwell-Wiechert (M-W) rheological model, the diagram of which is shown in Figure 6.

A stress relaxation function expressed by an Equation (2), after taking into account the five-parameter rheological model shown in Figure 6, can also be described in the following way, using Equation (3) [43].

(3) $$\sigma (t)=\varepsilon_{o}(E_{o}+E_{1}e^{-\frac{t}{t_{1}}}+E_{2}e^{-\frac{t}{t_{2}}})$$

where:

*ε_o_*—predetermined relative displacement, *ε*(*t*) *=ε_o_H*(*t*).

*H*(*t*)—Heaviside function.

*t*_1_, *t*_2_—time in relaxation; $t_{1}=\eta_{1}/E_{1};\;t_{2}=\eta_{2}/E_{2}$

*E_o_*, *E*_1_, *E*_2_, *η*_1_, *η*_2_—parameters of the selected rheological model.

**Figure 7:**
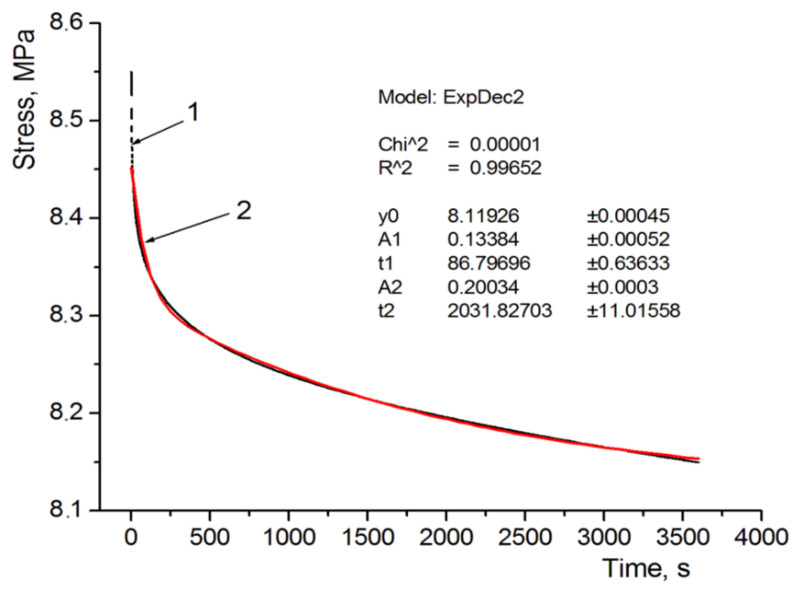
Approximation of the relaxation curve for sample D: 1—from the experiment; 2—relaxation function.

**Figure 8:**
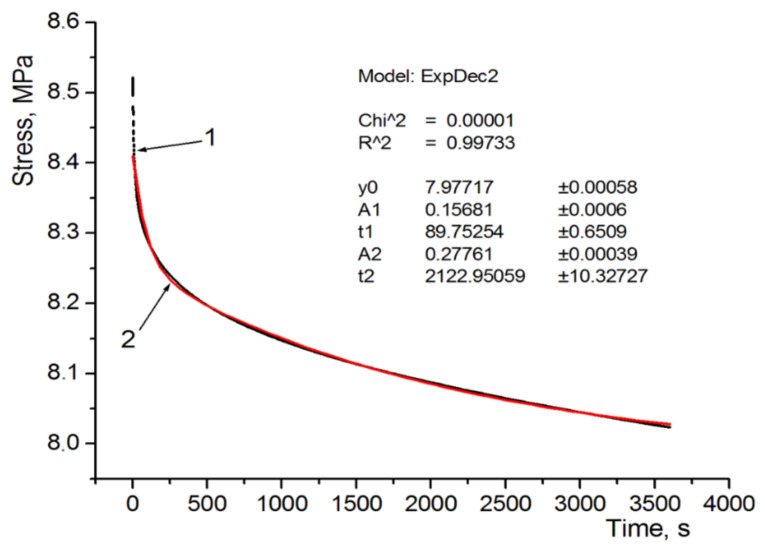
Approximation of the relaxation curve for sample E: 1—from the experiment; 2—relaxation function.

The coefficient values of the used rheological model are shown in Table 5. Sample graphs depicting stress approximation as a function of time are shown in Figure 7 and Figure 8. These figures apply to the analysis of specimens designated as D and E, i.e., with the same structure but a different internal filling. In the case of specimen D there is also a reference material, and in the case of specimen E—soft support material as structural filling. It can be seen that a 5-parameter model stress relaxation function approximation is a very accurate approximation of the results obtained directly from the universal testing machine, which is evidenced by calculated coefficients χ^2^ and R^2^.

Based on the conducted relaxation tests and M-W rheological model parameters resulting from approximation, it can be concluded that cellular structures made of polymer materials with the PJM method exhibit significantly different viscoelastic properties, especially in the case of additionally using soft support material. A comparison of the equivalent elasticity moduli values (*E_s_*) and the relaxation index (Δ*σ*) indicates that specimens with lower viscosity exhibit higher compressive stress reductions.

**Figure 9:**
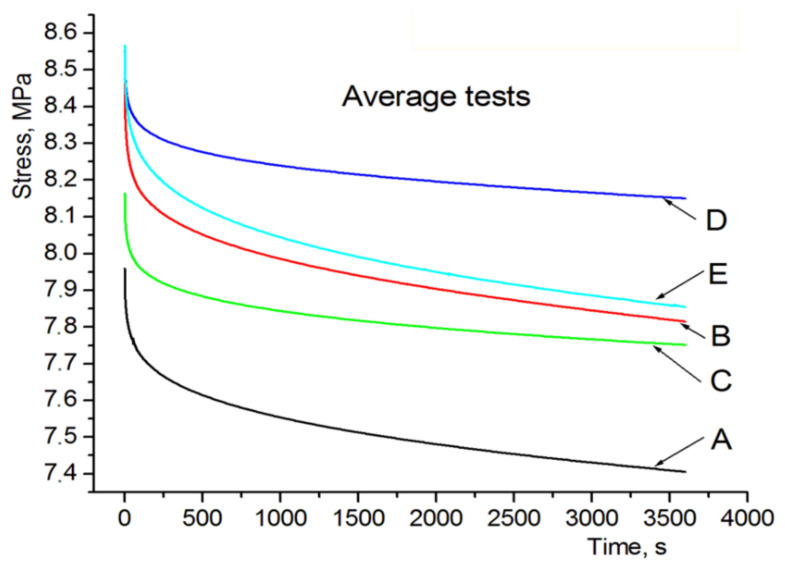
Averaged relaxation curves calculated for specimens A–E.

In order to compare the basic properties of polymer materials, i.e., FC 720, VW and FC 705, and the cellular structures made of them, Figure 9 lists averaged relaxation curves for specimens designated as A to E. The averaged relaxation curves for individual specimens were calculated as per function (3), after substituting average rheological parameter values specified in Table 5.

Figure 9 shows that relaxation characteristics for specimen C consisting of two parts made of model materials (FC 720, VW) is in between the characteristics for specimens A and B. Its waveform shows that the sandwich-type specimen C behaves more stable under applied compressive stress, compared to specimens A and B made of model materials.

Specimen D relaxation characteristic waveform would indicate that the application of an internal structure in the form of filled cylindrical channels increases the maximum relaxation stress, followed by faster stabilization of this stress, similarly to specimen C without an internal structure.

A comparison of the relaxation characteristics for specimens D and E indicates that the behavior of the internal structure in the form of perpendicular channels filled with the FullCure 705 soft material provides greater relaxation stress at the same relative strain.

### Microstructure Views

**Figure 10:**
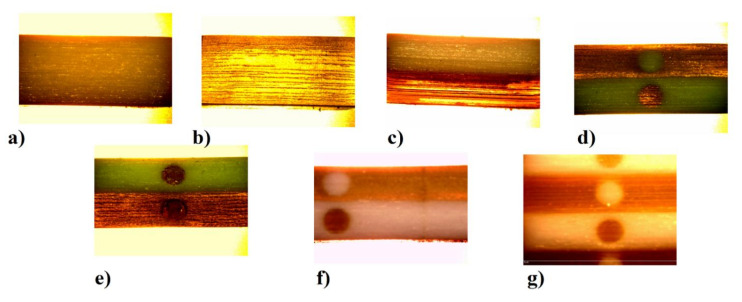
Lateral plane microscopic views of the samples, (**a**) samples—A, (**b**) samples—B, (**c**) samples—C, (**d**) sampes—D, (**e**) samples—E, (**f**) samples—F, (**g**) samples—G.

Figure 10 shows microscopic views of the specimens, with a 6× magnification, prepared using an Olympus microscope.

The photos of the views taken already at a low magnification clearly show the layered structure of the 3D printing PJM technology process. Microscopic images were taken after completed compression tests, which is particularly well visible in Figure 10e,f, where the central part of the specimens experienced strain, which might have been induced by the introduction of internal cellular structures of model and support material (Figure 10e) and opposing model materials (Figure 10f).

## 4. Discussion

After a qualitative analysis of the stress relaxation results shown in Figure 8 and in Table 4, it can be concluded that, in the case of specimen models made of the FullCure 720 model material (specimen A), the determined stress relaxation is the highest and amounts to 0.095, which means that the authors obtained the highest stress reduction over time, amounting to Δ*σ*–9.5%. An equally high stress relaxation drop as a function of time was obtained for specimen E, where Δ*σ*–0.085, which means that after a time t, the stress drop was over 8.5%. Specimen E is made of a material where the internal structures are filled with soft support material—FullCure 705, which justifies such a great drop of stress over time. The third-highest stress drop result as a function of time was obtained for specimen D, made entirely of VeroWhite. In this case the Δ*σ* strain reduction was 0.074. It can be seen that sandwich-type specimens (C, D, E and their extensions F and G) exhibit a clear tendency to change stress relaxation depending on the applied materials and the degree of complexity of their internal structures and filling. Specimen C exhibits stress relaxation Δ*σ*–0.073, which is a value close to that obtained for pure VeroWhite, however, in the case of expanding this structure with external structures, stress relaxation is reduced to a level of Δ*σ*–0.055 for specimen D (sandwich-type with internal channels filled alternately with model materials). It is clearly visible that such a combination of both materials, together with their opposing use in internal structures reduces stress relaxation and beneficially impacts their drop over time, i.e., increases resistance to continuous stress. This is an interesting conclusion, because it does not increase material consumption in any way, but rather just optimizes the structure. Specimen D, compared to specimen C, experienced stress reduction lower by almost 25%, which is a very good value, given the fact that the eternal shape and material consumption were not changed in any way. The only controlled aspect was the execution of the model as a cellular structure. It seems worth to analyses the comparison of the extension of specimens D and E, where the internal structures were made of soft support material. Specimen E is characterized by the relaxation coefficient Δ*σ*–0.085, which means that the application of soft materials as structural fillers greatly reduces resistance to load over time and, compared to specimen D, it experiences an increase of over 54%. It seems that soft model material can be used exactly when a quick response to loading the materials, in the form of dynamic stress drop, is expected. This tendency is clearly visible in Figure 4d,e. Analysis of the microscopic images did not show significant differences in the case of specimens D and E. Very brief initial results shown in the form of measurements for specimens F and G indicate the possibility of modular joining of cellular structure models and controlling the manufacturing process in order to obtain more complex structures with a set shape and desired mechanical and rheological properties. Analyzing the results of the research conducted for both FullCure 720 and VeroWhite model materials and supporting FullCure 705, and taking into account the literature review, it can be concluded that the obtained cell structures confirm the rheological relationships obtained in the works [42] and [41]. Development of the research with the shape of the cell structure and a new supporting material however, it does not provide a direct opportunity for a full comparative analysis. However, taking into account the results of the research presented in the [42] publication, it seems that it can be concluded that the production of cellular structures in PJM technology should take into account the direction of printing and avoid the variant of placing the CAD model on the 3D printer building platform at an angle of 45°. This variant, as shown by the literature review, is the least favorable the variant which is characterized by the greatest stress decrease as a function of time for the FullCure 720 material. This characteristic, however, does not apply to the VeroWhite material, which means that it can be assumed that the cellular structures obtained by combining the VeroWhite model material and the supporting FullCure 705 material can be resistant to the phenomenon of anisotropy depending on the direction of the print. The study of this phenomenon will be the subject of further research by the authors of the publication.

The aforementioned analysis of stress relaxation results for specimen models with a set structure shows a certain internal cellular structure experimental optimization process. Such optimization perfectly fits the realities of the new industrial revolution 4.0 and optimization methods, such as LEAN manufacturing and LPPD (Lean Process and Product Development), where the main goal is to manufacture structures with a minimum weight and maximum product requirement fulfilment.

## 5. Conclusions

When analyzing the presented results for specimens prepared using the PJM technology and subjected to relaxation tests, the following general conclusions can be drawn.

It is possible to optimize the LPPD and LEAN process by selecting appropriate model appearance using cellular structures and both models and support material.

Taking into account internal modular structures in solid-structure models transforming them into cellular-structure models enables obtaining models with controlled model material response and controlling the “self-control response model” process.

Future work of the research team will show how combining the aforementioned cellular structures impacts rheological properties and is it possible to control a technological process in order to obtain a model with a cellular structure and predicted material response. In addition, it is planned to include in the future research the impact of the print direction on the rheological properties and layer thickness.

## Figures and Tables

**Table 1:** Chemical composition of VeroWhite [43].

CAS	Components	Percentage
	VeroWhite	
Unavailable	Acrylicoligomers	30–50
Proprietary	Monomer	10–30
5888-33-5	2-propenoic acid, 1,7,7-trimethylbicyclo[2.2.1]hept-2-yl ester	10–30
87320-05-6	[2-[1,1-dimethyl-2[(1-oxoallyl)oxy]-5-ethyl-1,3-dioxan-5yl]methyl acrylate	0–10
154508-99-8	Epoxy acrylic oligomer	1–10
Proprietary	Photoinitiator	1–5

**Table 2:** Chemical composition of FullCure 720 [43].

CAS	Components	Percentage
	FullCure 720	
-	Acrylic monomer	<30
588-33-5	Isobornyl acrylate	<25
-	Phenol, 4,4’-(1-methylethylidene)bis-, polymer with (chloromethyl)oxirane, 2-propenoate	<15
-	Phosphine oxide, phenylbis(2,4,6-trimethylbenzoyl)-	<2
52408-84-1	Acrylic acid ester	<0.3

**Table 3:** Chemical composition of FullCure 705 [43].

CAS	Components	Percentage
	FullCure 705	
-	Poly(oxy-1,2-ethanediyl), α-(1-oxo-2-propenyl)-ω-hydroxy-	<50
57-55-6	1,2-Propylene glycol	<35
25322-68-3	Polyethylene glycerol	<30
56-81-5	Glycerin	<25
-	Phosphine oxide, phenylbis(2,4,6-trimethylbenzoyl)-	<0.5
52408-84-1	Acrylic acid ester	<0.3

**Table 4:** Stress relaxation indices for all specimens.

Stresses Relaxation Indicators—Mean Value	Specimen Type						
	A	B	C	D	E	F	G
Δσ	0.095	0.074	0.073	0.055	0.085	0.033	0.039
SD	0.049	0.005	0.016	0.009	0.025	0.005	0.013

**Table 5:** Parameters of the rheological model.

Specimen	*E_o_*, MPa	*E*_1_, MPa	*E*_2_, MPa	*η*_1_, MPa⋅s	*η*_2_, MPa⋅s	*E_S_*, MPa
A	72.945	2.592	4.384	253.199	8449.591	79.921
B	77.399	1.923	3.527	179.174	30942.076	82.849
C	76.067	2.019	3.412	134.879	6878.735	81.498
D	80.614	1.493	2.829	134.421	5850.091	84.936
E	76.251	2.221	4.446	224.879	9246.132	82.918
F	21.564	0.264	0.381	22.573	779.498	22.209
G	20.497	0.280	0.442	24.716	886.282	21.219

## Data Availability

The data created in this study are fully depicted in the article.
